# Identity of *Squalius* (Actinopterygii, Cyprinidae) from Istra Peninsula in Croatia (Adriatic Sea basin)

**DOI:** 10.3897/zookeys.53.472

**Published:** 2010-08-27

**Authors:** Primoz Zupancic, Milorad Mrakovcic, Zoran Marcic, Alexander M. Naseka, Nina G. Bogutskaya

**Affiliations:** 1 Dolsko 14, 1262 Slovenia; 2 Department of Zoology, Faculty of Science, University of Zagreb, Rooseveltov Trg 6, 10000 Zagreb, Croatia; 3 Zoological Institute, Russian Academy of Sciences, Universitetskaya Emb. 1, St. Petersburg, 199034, Russia

**Keywords:** freshwater fish distribution, Cyprinidae, Istrian chub, Squalius, morphology, Adriatic Sea basin, Istra Peninsula, Slovenia, Croatia

## Abstract

A chub of previously ambiguous identity from the Boljunscica and Pazincica rivers (south-eastern Istra Peninsula) was studied and compared with geographically close Squalius squalus, Squalius zrmanja, and Squalius janae recently described from the Dragonja River drainage in the Adriatic Sea basin in Slovenia. It was shown that the chub from the south-eastern Istra Peninsula differs from all know species of Squalius but one: Squalius janae. Three samples examined from Boljunscica and Pazincica rivers and Squalius janae from its type locality, Dragonja River, show the following characters typical for the latter species: a long head (the head length 27–32% SL); a pointed conical snout with a clearly projecting upper jaw; a long straight mouth cleft, the lower jaw length (39–45% HL) exceeding the caudal peduncle depth; a large eye; commonly 9? branched anal-fin rays; commonly 44 total vertebrae (24+20 or 25+19); bright silvery colouration, scales easily lost; iris, pectoral, pelvic and anal fin pigmentation with yellow shades. The data on the distribution of Squalius chubs in the northern Adriatic basin support the assumption that the range of Squalius janae is determined by the geology of the Trieste Flysch Basin and the Pazin Flysch Basin forming the base of the Istra Peninsula. The distribution pattern of this species does not support a simple model of fish dispersal and a complete connectivity within the whole Palaeo-Po historical drainage. Indeed, it indicates a disrupted surface palaeohydrography that was heavily fragmented by karstification in the whole Dinaric area.

## Introduction

Istra [Istria, formerly Histria], is the largest peninsula in the Adriatic Sea. It is located in the northern Adriatic between the Gulf of Trieste and the Bay of Kvarner, and shared by three countries: Croatia, Slovenia and Italy. The largest portion, Hrvatska Istra [Croatian Istria] lies in Croatia. Larger rivers of the western cost of the Istra Peninsula are (from north to south) Osapska Reka [Osbo], Rižana [Risano], Badaševica [Kornalunga] (with a reservoir called Vanganel Lake), Dragonja with its largest tributary Pinjevec [Rokava], and Mirna [Quieto], which is the largest river in Istra. The Butoniga River, a tributary of the Mirna, was dammed in 1989 in its upper part and now flows into a reservoir called Butoniga Lake. In the south-east from the Mirna, Pazinčica [Foiba, Fojba] River flows for 16.5 km and in the Pazinska Jama [Pazin Cave] becomes a subterranean river which used to flow underground near Beram, Kringa and Dvigrad to the sea forming the Lim Valley; its former estuary is the Lim Bay [Limski Kanal]. Rivers Raša [Arsia] (flowing into the Raški Zaljev [Raski Inlet, Porto d’Arsia] and Boljunšćica [Boljunčica] (used to flow into the Plumin Luka Inlet or Plumin Bay) go southwards along the western slope of the Učka mountains [Monte Maggiore] and the Ćićarija [Ciceria] mountain range. At present, the Boljunšćica and Raša drainage systems in the region of Čepić Polje (ca. 45°12'N; 14°08'E) are interconnected by a number of irrigational canals and canalised streams. Till 1932 Čepić Polje had been a lake, which is now drained.

Chubs were known to occur in the Istra Peninsula, and historically they were identified as Squalius cavedanus (Bonaparte, 1838)in Osapska Reka and Rižana (Heckel and Kner 1858), as Leuciscus cephalus cabeda Risso, 1827in Rižana, Pazinčica, and Mirna (Gridelli 1936), as Leuciscus cephalus albus Bonaparte, 1838 in Čepić Lake [Lago d’Arsa nell Istria, Cepich-See] (Gridelli 1936: fig. 13), as Squalius cephalus (Linnaeus, 1758) in Badaševica [Kornalunga] (Porečnik 1958), as Leuciscus cephalus albus in Dragonja with Pinjevec (Povž and Sket 1990, Povž 2002) and in Butoniga Lake (Mrakovčić et al. 2000), as Leuciscus cavedanus in Mirna and Pazinčica(Mrakovčić et al. 2006), and as Squalius squalus (Bonaparte, 1837) in the whole of Istra (Kottelat and Freyhof 2007). Leiner and Popović (1984) reported Leuciscus svallize (Heckel & Kner, 1858) and Leuciscus cephalus albus for all Istrian rivers and reservoirs. Povž and Sket (1990) had doubts which subspecies (Leuciscus cephalus cabeda or Leuciscus cephalus albus) inhabits the numerous Adriatic rivers southwards from Dragonja. Leiner et al. (1995) reported Leuciscus cephalus albus from Čepić Lake and Pazinčica based on literature sources and from Mirna (with its tributary Bracan [Bračana]), Butoniga River and Butoniga Lake, Pazinčica, Raša, Boljunšćica, Letaj Reservoir, Dragonja with its tributary Pinjevec, and Vanganel Lake based on their own data. At the same time they mentioned (Leiner et al. 1995) Leuciscus cephalus cabeda as occurring in Rižana, Pazinčica, Mirna, and Badaševica with tributaries and Leuciscus cephalus in Mirna and Boljunšćica rivers based on literature sources. Besides, Leuciscus leuciscus (Linnaeus, 1758)is reported as occurring in Pazinčica and Mirna based on literature data (Leiner et al. 1995), and Leuciscus svallize in Mirna, Pazinčica, Raša, Boljunšćica, Letaj Reservoir, Rakov Potok Reservoir at Cerovlje (Leiner et al. 1995). Bogutskaya and Zupančič (1999) had no specimens from the Istra Peninsula available for examination and assumed that the reports of Leuciscus svallize might refer to either Leuciscus zrmanjae (Karaman, 1928) or Leuciscus illyricus (Heckel & Kner, 1858).

Zupančič (2008) in his review of endemic species of the Adriatic basin provided a list of forms he considered to be undescribed species, and gave the chub from Dragonja, Mirna, Pazinčica, Boljunšćica and Raša rivers as Squalius sp. 1 (Zupančič 2008). The Dragonja material has been recently described as a new species Squalius janae Bogutskaya and Zupančič, 2010 which is distinguished from other species of the genus Squalius in the Adriatic basinby a long head, a pointed conical snout, a straight oblique mouth cleft, a long lower jaw length, a large triangular 5th infraorbital, commonly 44–47 total lateral line scales, commonly 9½ branched anal-fin rays, commonly 24+20 or 25+19 vertebrae, strong silvery tint in colouration, iris, pectoral, pelvic and anal fin pigmentation with yellow shades, and scales easily lost. Bogutskaya and Zupančič (2010) showed that a chub from northern Istra Peninsula: Osapska Reka, Rižana, Malinska and Mirna (except for Dragonja) belongs to Squalius squalus

The purpose of the present article is to describe the chub from Pazinčica and Boljunšćica rivers (south-eastern Istra), compare it with Squalius janae and Squalius squalus, and decide on its identity.

## Material and methods

Measurements were taken point to point to the nearest 0.1 mm following Bogutskaya and Zupančič (1999). The standard length (SL) was measured from the tip of the upper jaw to the end of the hypural complex. The length of the caudal peduncle was measured from behind the base of the last anal-fin ray to the end of the hypural complex, at mid-height of caudal-fin base. Head length (HL) and interorbital width were measured including the skin fold. Postdorsal length was measured from the dorsal-fin insertion to the posterior end of the hypurals, and the dorso-hypural distance was taken from the origin of the dorsal fin to the posterior end of the hypurals. A further character was added from Doadrio et al. (2007): a point where the dorso-hypural distance, which is taken from the dorsal-fin insertion to the end of the hypural complex, falls when measured forward. The last two branched rays articulated on a single pterygiophore in dorsal and anal fins were counted as “1½”. Total lateral line scale counts include all pored scales, from the first one just behind the posttemporal bone to the posteriormost one located on the bases of the caudal-fin rays. Osteological characters were examined from radiographs.

Abbreviations used: CBDCollection of G.A.C. Balma and G.B. Delmastro, Torino; NMWNaturhistorisches Museum Wien; SMNHSlovenian Museum of Natural History, Ljubljana; PZCCollection of P. Zupančič, Dolsko (Slovenia); ZISPZoological Institute, Russian Academy of Sciences, St. Petersburg; CNHMCroatian Natural History Museum, Zagreb.

### Material from the south-eastern Istra Peninsula

Squalius janae. NMW 3077–080, 7, 160.1–186.8 mm SL; Croatia: Čepić Lake; Steindachner don., V.1900. – NMW 49229, 16, 120.1–168.4 mm SL; same locality and donator, 1900 v.2a. – NMW 49250, 1, 222.8 mm SL; Croatia: Čepić Lake; coll. Krauss, August 1877. – PZC 406, 29, 49.5–133.2 mm SL; Croatia: Boljunšćica River at Katun Boljunski, ca. 45°17'N; 14°08'E; coll. Zupančič, 4 September 2004. – PZC 457, 5, 74.5–160.6 mm SL; same locality and collector; 3 September 2007. – PZC 415, 16, 50.8–118.0 mm SL; Croatia: Pazinčica River, ca. 45°14'N; 13°55'E; 13 July 2000, coll. Mrakovčić; CNHM 5005, 5002, 6021 and 5164, 4, 96.6–113.0 mm SL; Croatia: Istra Peninsula [no exact locality, probably Pazinčica]; no date, leg. Leiner.

### Comparative Material

Squalius janae. SMNH 207, holotype 183.7 mm SL; Slovenia: Dragonja River about 2 km upstream from bridge on road from Župančiči, 45°28'N; 13°46'E; coll. Zupančič, 1 August 2008. –PZC 475, 6 paratypes, 85.9–174.9 mm SL; same data as holotype. – PZC 452, 6 paratypes, 74.9–132.5 mm SL; Slovenia: Dragonja River at bridge on road from Župančiči, 45°28'15"N; 13°45'11"E; coll. Zupančič, 24 November 2007. – PZC 453, 6 paratypes, 66.2–157.6 mm SL; same locality; coll. Zupančič, 17 May 2009. – PZC 454, 19 paratypes, 85.8–158.0 mm SL; Slovenia: Dragonja River downstream from confluence with Pinjevec [Rokava], 45°28'30"N; 13°44'31"E; coll. Zupančič, 10 April 2009. – PZC 455, 7 paratypes, 68.4–212.7 mm SL; Slovenia: Dragonja River south from Koštabona, 45°28'20"N; 13°44'11"E; coll. Zupančič, 10 April 2009. – PZC 456, 8 paratypes, 111.2–190.0 mm SL; Slovenia: Dragonja drainage: Pinjevec River [Rokava] at Župančiči, 45°29'N; 13°46'E; coll. Zupančič, 1 February 2007. – PZC 476, 4 paratypes, 80.4–120.0 mm SL; same locality and collector; 24 November 2007. – ZISP 54690, 11 paratypes, 88.5–149.3 mm SL; same locality as PZC 455; coll. Zupančič and Naseka, 5 July 2008.

Squalius squalus (samples are presented below in geographical order, from north-west to south-east). NMW 48920, 1 [probable syntype] of Squalius tyberinus Bonaparte, 1841, 187 mm SL; Italy: Tyber; “[C.L. Bonaparte], 1844.VI.19”. – NMW 49189, 5, 65.7–193.8 mm SL; Treviso; “[Tausch], 1844.V”. – NMW 49259, 4, 150.6–195.5 mm SL; Slovenia: Rižana [Risano, 1848.I.10]. – PZC 287 (from CBD–F1996/44384–44391), 8, 80.5–155.4 mm SL; Italy: Po drainage: stream Orco, 100 m from confluence with Po River at Chivasso, 45°12'N; 07°53'E; coll. Balma and Delmastro, 18 September 1991. – PZC 288 (from CBD–F2057/45572–45576), 5, 75.5–140.5 mm SL; Italy: Po drainage: Torrente Malone, about 1 km upstream of the bridge of Lombardore, 45°14'N; 07°44'E; Balma and Delmastro, 12 May 1992. – PZC 458, 4, 140.0–185.0 mm SL; Slovenia: Soča drainage: Nadiža [Natisone] River at Podbela, 46°14'30"N; 13°27'30"E; coll. Zupančič, 2 May 2007. – PZC 459, 3, 139.3–185.0 mm SL; Slovenia: Soča drainage: Idrija [Idria] River at Velendol, 46°05'N; 13°34'E; P. Zupančič, 2 May 2007. – PZC 460, 5, 107.0–145.0 mm SL; Slovenia: Soča drainage: Idrija system, Koncnar River, 45°59'N; 13°22'E; coll. Zupančič, 8 December 2007. – PZC 461, 12, 120.0–220.0 mm SL; Slovenia: Soča drainage: Idrija system, Birša River at Dolnje Cerovo, 45°58'N; 13°33'E; P. Zupančič, 26 April 2007. – PZC 462, 4, 140.0–215.0 mm SL; Slovenia: Soča drainage: Vipava [Vipacco] system, Močilnik River at Slap; 45°50'N; 13°56'E; P. Zupančič, 26 April 2007. – PZC 463, 7, 135–205 mm SL; Slovenia: Soča drainage: Vipava system, River Močilnik at Vipava, 45°50'08"N; 13°57'E; coll. Zupančič and Naseka, 6 August 2009. – ZISP 54687, 13, 107.8–208.2 mm SL; Slovenia: Soča drainage: Vipava system, Branica River at Steske, 45°52'30"N; 13°46'E; coll. Zupančič and Naseka, 5 July 2008. – PZC 465, 2; Slovenia: Soča drainage: Vipava system, Vrtovinšček River at Fuzine, 45°53'40"N; 13°48'40"E; coll. Zupančič and Naseka, 5 July 2008. – PZC 466, 14, 98.5.0–190.0 mm SL; Slovenia: Soča drainage: Vipava system, Vogršček River at Vogrsko; 45°50'N; 13°56'E; coll. Zupančič, 1 December 2007. – PZC 467, 9, 97.0–155 mm SL; Slovenia: Osapska Reka River (flows into Miljski Zaliv east of Muggia) at Osp, 45°33'N; 13°52'E; coll. Zupančič, 1 February 2007. – ZISP 54689, 7, 96.7–165.5 mm SL; same locality as PZC 467, coll. Zupančič and Naseka, 10 July 2008. – ZISP 54689, 6, 88.0–190.6 mm SL; Slovenia: Rižana River (flows into Miljski Zaliv at Koper [Capadistria]) at Dekani, 45°32'30"N; 13°48'E; coll. Zupančič and Naseka, 5 July 2008. – PZC 468, 2, 175, 182 mm SL; Croatia: Malinska River (endorheic, south from Dragonja) at Koromači Buskini, 45°27'N; 13°50'E; coll. Zupančič, 28 March 2009. – PZC 469, 4, 129.5–172.7 mm SL; Croatia: Mirna River at Livade, 45°21'N; 13°50'E; coll. Zupančič, 4 August 2005. – PZC 470, 7, 112.5–176.5 mm SL; Slovenia: Reka [Bračana] River, tributary of Mirna, at Olika, 45°27'40"N; 13°53'40"E; coll. Zupančič, 12 November 2008.

Lists of examined specimens of Squalius cephalus, Squalius cf. squalus, Squalius zrmanjae, Squalius svallize, Squalius illyricus, Squalius prespensis, and Squalius orientalis can be found in Bogutskaya and Zupančič (1999, 2010).

**Figure 1.**
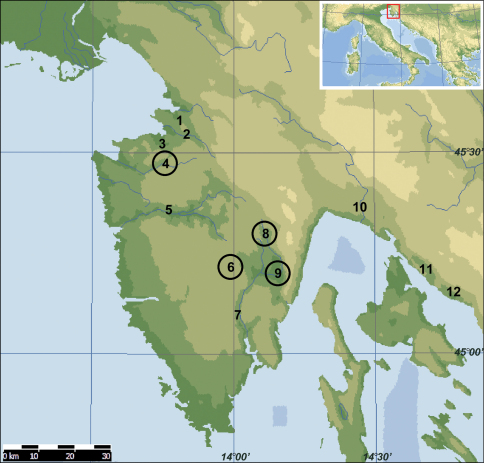
Map showing the main localities mentioned in the text: **1** Osapska Reka **2** Rizana **3** Badasevica and Vanganel Lake **4** Dragonja with Pinjevec **5** Mirna with Reka and Butoniga **6** Pazincica **7** Rasa **8** Boljunscica **9** Cepic Polje (formerly Cepic Lake) **10** Rjecina **11** Dubracina and Tribalj Reservoir **12** Ricina. Localities where Squalius janae is recorded are circled.

A map showing the localities of samples from the Istra Peninsula is given in Fig. 1.

## Results

### Description of specimens from Boljunšćica and Pazinčica rivers.

**Figure 2.**
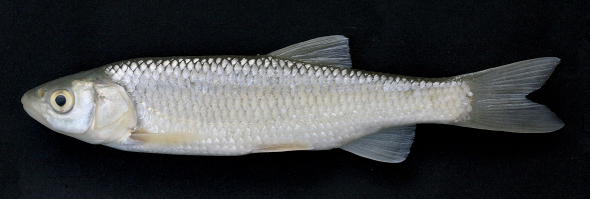
Squalius janae, live specimen. PZC 457, 160.6 mm SL, Croatia: Boljunscica.

**Figure 3.**
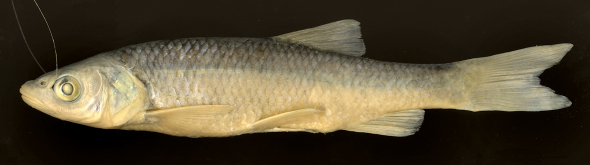
Squalius janae NMW 49229, 147.5 mm SL, Croatia: former Cepic Lake.

**Figure 4.**
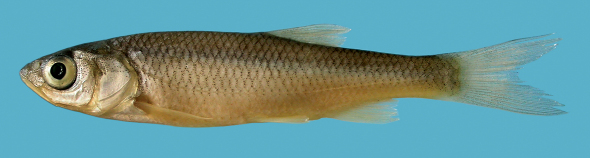
Squalius janae PZC 406, 50.0 mm SL, Croatia: Boljunscica.

**Figure 5.**
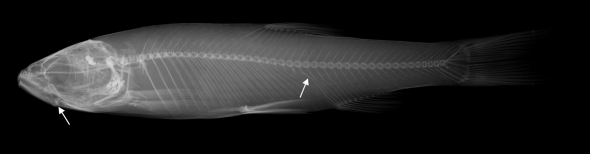
Radiograph of Squalius janae, PZC 406, 133.2 mm SL, Croatia: Boljunscica, showing 24+20 total vertebrae (arrow shows first caudal vertebra) and lower jaw length (arrow shows posterior end of lower jaw).

Morphometric data of 30 specimens from the Boljunšćica and Pazinčica rivers are given in Table 1, and the general appearance can be seen from Figs 2–5.

The body is elongate, depth at the dorsal-fin origin is 21–24% SL. The head is long, its length, 27–31% SL, is greater than the body depth being 113–131% of the latter, and exceeds the caudal peduncle depth by a factor of 2.6–3.2. The upper head profile is almost straight, the snout is pointed, and the preorbital part of the head is almost triangular in lateral view. The mouth is subterminal (Figs 2, 3) or almost terminal though never clearly terminal even in smaller specimens (Fig. 4), and the upper jaw is clearly projecting beyond the lower jaw. The mouth cleft is straight, oblique, and the lower jaw-quadrate junction is commonly well visible forming a distinctive obtuse angle (Figs 2–5); the lower jaw-quadrate junction is on the vertical through the middle of the eye. The lower jaw length, 39–44% HL, exceeds the caudal peduncle depth by a factor of 1.0–1.3, commonly 1.1–1.2. The length of the lower jaw is always greater than both the operculum depth (being 106–116% of the former) and the interorbital width. The eye is large, its horizontal diameter being 20–25% in HL, and 54–71% in interorbital width. The eye diameter negatively correlates with the fish size as it can be seen from comparisons of larger and smaller specimens (Figs 2–3 and 4), for example, it is 26–28% in HL in specimens of 50–60 mm SL and 20% in HL in a specimen of 222 mm SL (NMW 49250) though we did not perform a statistic comparison.

The dorsal fin has 3 simple and 8½ branched rays in all specimens. Its outer margin is straight. The dorsal fin is located slightly behind the end of the pelvic-fin base. The dorso-hypural distance falls when measured anteriorly in the posterior half of the eye, rarely at the posterior eye margin. The dorsal fin is high, its depth being 16–19% in SL. The anal fin has 3 simple and 9½ branched rays; 8½ branched rays were found only in two specimens. The anal-fin outer margin is slightly to markedly convex.

The total number of gill rakers in the outer row on the first left gill arch is 8(5), 9(22) or 10(3). Pharyngeal teeth (in five specimens examined) are 2.5–5.2, hooked and serrated. The number of total lateral line scales is 43(1), 44(2), 45(12), 46(11), 47(9) or 48(3). Scales on flanks are easily lost in both live and preserved specimens. The total vertebrae including four Weberian vertebrae and the last complex centrum are 44 in 10 specimens examined; the number of abdominal vertebrae (including intermediate ones; precaudal vertebrae auctorum) is 24(5) or 25(5); the number of predorsal vertebrae (anterior to the first dorsal pterygiophore) is 15(9) or 16(1); intermediate vertebrae are five in all specimens; the number of caudal vertebrae is 19(5) or 20(5). The vertebral formulae are 25+19 (5) or 24+20 (5) (Fig. 5).

In live specimens, the overall colouration has a strong silvery tint, and the back is only slightly darker than the flanks and the belly. No conspicuous dark reticulated pattern has been seen in live specimens. The iris, anal and pectoral fin pigmentation has yellow shades, and is never bright (Fig. 2). Formalin fixed and ethanol stored specimens keep silvery-grey colouration with no brownish or bronze shades; yellow pigments are often lost in preserved specimens. Pigmentation on scales shows a reticulate pattern with comparatively fewer pigment dots along the outer scale margins. A concentration of pigment on scale pockets forming dark vertical spots is commonly visible in larger specimens (Fig. 2) though not pronounced in a small specimen (50 mm SL, Fig. 3).

The Pazinčica and Boljunšćica specimens and the historical sample from Čepić Lake examined in this study are thus identified as Squalius janae based on the diagnostic characters of the latter (Bogutskaya and Zupančič 2010), see Table 1 and Figs 2–5.

### Comparative remarks.

**Figure 6.**
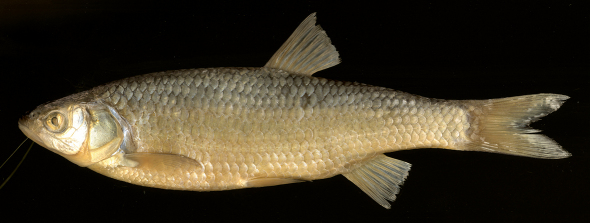
Squalius squalus NMW 49259, 195.5 mm SL, Slovenia: Rizana.

The Pazinčica and Boljunšćica specimens and the Čepić Lake historical sample differ from Squalius squalus in general and from specimens from Osapska, Rižana (Fig. 6), Malinska and Mirna rivers in the northern Istra Peninsula in particular (see Bogutskaya and Zupančič 2010) by having a shallower body, the body depth at the dorsal-fin origin being 21–25% SL (means 22.5 and 23.4 in the Boljunšćica and Pazinčica samples, respectively) while in Squalius squalus the body is deeper, 23–28% SL (mean 25.4 in the Osapska sample, from Bogutskaya and Zupančič 2010). The head is longer in Squalius janae than in Squalius squalus: in Squalius janae the head length is 28–32% SL (means 28.2 and 29.1 in the Boljunšćica and Pazinčica samples, respectively) in contrast to 25–29% SL in Squalius squalus (mean 26.5 in the Osapska sample). The depth of the caudal peduncle is 2.6–3.2 in HL in Squalius janae (means 2.7 and 2.8 in the Boljunšćica and Pazinčica samples, respectively) while in Squalius sqaulus the ratio is 2.2–2.8. The length of the lower jaw clearly exceeds the caudal peduncle depth in most specimens from the Boljunšćica and Pazinčica samplesthat is typical of Squalius janae,while it is about equal to or less than the caudal peduncle depth in Squalius squalus (Fig. 6). Additionally, the Boljunšćica and Pazinčica specimens have a straight oblique mouth cleft with a distinct angle at the lower jaw-quadrate junction, similar to the condition seen in Squalius janae and in contrast to Squalius squalus, which has a rounded snout and a shorter mouth cleft, curved in its anterior part and in a more horizontal position (Fig. 6; Bogutskaya and Zupančič 2010: figs 4a, b, 9). The Boljunšćica and Pazinčica specimens are further distinguished from Squalius squalus by vertebral counts having 44 total vertebrae (Fig. 4), vertebral formulae being 24+20 or 25+19 vs. commonly 43 and 25+18, and a silvery colouration vs. darker colouration with brownish or slight bronze tones in Squalius squalus.

Squalius zrmanjae (Zrmanja and Krka Rivers) is another species geographically close to Squalius janae. A morphological comparison between the two species and between Squalius janae and Squalius illiricus distributed in Cetina and Krka can be found in Bogutskaya and Zupančič (2010), and some distinguishing characters are summarised in the key below.

**Table 1:** Morphometric data of Sualius janae from Dragonja, Boljunšćica and Pazinčica rivers. Diagnostic characters of Squalius janae are given in boldface.

	Dragonja (PZC 475, 452-54, ZISP 54690, holotype SMNH 207, n=42)				Boljunšćica (PZC 406, 457, n=20)				Pazinčica (PZC 415, n=10)			
	Min	Max	Mean	SD	Min	Max	Mean	SD	Min	Max	Mean	SD	n	SD
SL, mm	63.3	190.0	80.0	160.6	75.3	118.0	
**Body depth at dorsal-fin origin (% SL)**	**20.4**	**26.1**	**23.16**	**1.09**	**20.9**	**23.6**	**22.48**	**0.87**	**22.9**	**25.2**	**23.41**	**0.86**
Depth of caudal peduncle (% SL)	9.7	11.0	10.46	0.38	9.8	10.9	10.37	0.38	9.3	10.8	10.37	0.48
Depth of caudal peduncle (% length of caudal peduncle)	47.9	58.8	54.31	3.32	47.47	58.30	53.09	3.21	49.1	57.4	54.43	2.64
Predorsal length (% SL)	55.4	59.0	57.04	1.06	55.6	58.4	56.61	0.87	57.1	59.3	58.00	0.83
Postdorsal length (% SL)	32.6	36.2	34.17	0.95	34.3	35.7	35.11	0.47	32.6	35.4	33.94	1.12
Preanal length (% SL)	71.2	76.0	74.05	1.30	71.0	74.4	72.60	0.96	70.6	73.7	72.56	1.04
Pectoral – pelvic-fin origin length (% SL)	24.3	29.0	26.76	1.20	24.4	26.8	25.70	0.75	25.5	26.6	26.20	0.37
Pelvic – anal-fin origin length (% SL)	19.3	22.7	21.32	0.85	19.7	22.8	21.38	0.98	20.2	22.3	21.43	0.86
Length of caudal peduncle (% SL)	18.0	21.1	19.31	0.89	18.1	21.0	19.58	0.83	18.5	19.4	19.05	0.32
**Dorsal-fin base length (% SL)**	**10.1**	**12.1**	**10.98**	**0.60**	**9.9**	**11.6**	**10.92**	**0.42**	**10.0**	**11.0**	**10.65**	**0.35**
Dorsal fin depth (% SL)	16.3	20.2	18.09	1.32	17.4	19.4	17.91	0.57	16.1	19.1	17.62	1.04
Anal-fin base length (% SL)	9.7	11.0	10.48	0.66	10.0	11.4	10.79	0.46	9.9	11.1	10.46	0.51
Anal fin depth (% SL)	12.5	16.1	14.28	0.98	13.3	15.8	14.63	0.64	13.4	15.0	13.99	0.61
Pectoral fin length (% SL)	18.8	22.0	20.17	0.94	18.8	21.6	19.92	0.81	18.5	20.7	19.83	0.78
Pelvic fin length (% SL)	15.2	17.6	16.21	0.74	15.4	17.1	16.36	0.57	14.3	16.5	15.69	0.79
**Head length (% SL)**	**28.6**	**31.6**	**29.44**	**0.80**	**27.1**	**29.6**	**28.15**	**0.64**	**28.1**	**30.2**	**29.09**	**0.77**
**Head length (% body depth)**	**111.0**	**137.2**	**123.07**	**6.18**	**118.08**	**131.03**	**125.33**	**3.69**	**112.6**	**129.6**	**119.33**	**5.40**
Head depth at nape (% SL)	17.3	18.8	18.07	0.51	16.14	18.40	17.36	0.70	17.0	18.3	17.53	0.46
**Head depth at nape (% HL)**	**59.1**	**64.2**	**61.39**	**1.63**	**58.2**	**64.6**	**61.67**	**1.92**	**58.9**	**62.6**	**61.29**	**1.28**
Head depth through eye (% HL)	42.3	47.5	44.55	1.43	41.6	48.1	44.61	1.84	42.7	46.3	44.44	1.54
Maximum head width (% SL)	14.9	18.9	15.98	1.07	14.13	15.34	14.68	0.36	14.9	15.8	15.34	0.38
Maximum head width (% HL)	50.8	60.3	54.29	1.64	50.4	54.1	52.15	1.10	51.3	54.2	52.74	1.23
Snout length (% SL)	8.4	9.7	8.97	0.42	7.81	9.00	8.32	0.39	8.0	9.1	8.54	0.40
Snout length (% HL)	28.1	32.4	30.46	1.19	27.9	31.6	29.57	1.25	27.5	30.6	29.36	0.96
Eye horizontal diameter (% SL)	5.6	7.8	6.56	0.50	5.60	6.85	6.23	0.42	6.2	7.2	6.76	0.44
Eye horizontal diameter (% HL)	19.3	25.2	22.27	1.60	19.7	23.8	22.13	1.30	21.0	24.8	23.24	1.41
**Eye horizontal diameter (% interorbital width)**	**53.7**	**69.5**	**60.57**	**5.51**	**54.22**	**65.59**	**60.14**	**3.42**	**55.6**	**71.1**	**63.13**	**5.82**
Postorbital distance (% HL)	48.2	54.6	50.87	1.67	49.5	57.3	53.46	1.90	50.5	53.8	51.85	1.13
Interorbital width (% HL)	33.4	41.7	36.87	1.87	34.2	40.8	36.83	1.74	34.8	40.6	36.94	1.97
Length of upper jaw (% HL)	31.3	36.6	34.73	1.31	31.3	36.1	33.70	1.28	31.5	34.4	33.30	1.09
Length of lower jaw (% SL)	11.3	13.2	12.24	0.54	11.13	12.78	11.87	0.47	11.5	12.7	12.31	0.39
Length of lower jaw (% HL)	39.2	44.8	41.58	1.66	39.0	44.0	42.17	1.40	41.1	43.6	42.33	1.12
Length of lower jaw (% operculum depth)	106.7	126.7	115.38	5.81	105.95	113.54	109.67	2.42	106.6	115.8	111.08	3.18
Depth of operculum (% HL)	33.1	39.3	36.09	1.75	36.8	39.4	38.44	0.75	37.0	39.2	38.12	0.78
*Ratios*	
Interorbital width/eye horizontal diameter	1.4	2.0	1.65	0.16	1.5	1.8	1.67	0.10	1.4	1.8	1.60	0.15
Snout length/eye horizontal diameter	1.2	1.6	1.35	0.12	1.2	1.6	1.34	0.09	1.1	1.4	1.27	0.10
Head depth at nape/eye diameter	2.4	3.2	2.74	0.19	2.6	3.1	2.79	0.15	2.4	2.8	2.60	0.15
**Head length/depth of caudal peduncle**	**2.6**	**3.0**	**2.82**	**0.10**	**2.6**	**2.9**	**2.72**	**0.85**	**2.6**	**3.2**	**2.81**	**0.19**
Length of caudal peduncle /depth of caudal peduncle	1.7	2.1	1.86	0.13	1.7	2.1	1.88	0.12	1.7	2.0	1.83	0.9
**Length of lower jaw/caudal peduncle depth**	**1.1**	**1.3**	**1.17**	**0.06**	**1.0**	**1.2**	**1.15**	**0.07**	**1.1**	**1.3**	**1.19**	**0.06**
Pectoral fin length/pectoral – pelvic-fin origin distance	0.7	0.9	0.76	0.06	0.7	0.9	0.78	0.04	0.7	0.8	0.76	0.04
Predorsal length/head length	1.8	2.0	1.95	0.05	1.9	2.1	2.01	0.04	1.9	2.1	1.99	0.05

## Distribution of chubs in Istra Peninsula

The known range of Squalius janae (Fig. 1) includes Dragonja River (type locality) draining to the west, Pazinčica River that used to flow westwards but at present terminates in a cave at Pazin, and Boljunšćica River that used to flow southwards but now ends in canals in an area that was formerly Čepić Lake. We do not have materials from Raša River that is adjacent to the Boljunšćica and is now connected by canals to the latter. There are no historical samples known to us to decide upon the identity of the native Raša chub.

In rivers of north Istra (Osapska Reka, Rižana, Malinska, and Mirna) occurs a chub, which was identified by Bogutskaya and Zupančič (2010) as Squalius squalus, a species distributed widely further north- and westwards in the Adriatic Basin. Thus, in the north-west of the Istra Peninsula the range of Squalius janae is interrupted by Mirna River, which is now inhabited by Squalius squalus This may be explained as a historic indication of a direct connection of the Mirna to the Palaeo-Po system but a relatively recent introduction cannot be excluded either. To the east, Rječina River is the stream closest to Istra Peninsula, flowing into the Adriatic Sea at Rijeka. No chubs (Squalius) are known from this river (Šprem 2006). Further southwards, there is the Dubračina, a small, now endorheic river system, with the Tribalj Reservoir, which is inhabited by a probably introduced Alburnus, a Rutilus, and introduced Cyprinus carpio. There is a chub in Dubračina (Zupančič’s data) though no specimens were collected to check their identity. There are no published data indicating that a chub occurs in Ričina, a river located further down and flowing into the Adriatic at Novi Vinodolski. There are no native Squalius species in the entire Lika region endorheic drainages (Lika, Jadova, Otuča and some others) that lie south and west from Velika Kapela and Mala Kapela mountains southwards to Gračac. Squalius cephalus was introduced from the Danube to the Lika River (specimens in PZC). Further southwards, the only species of Squalius Squalius zrmanjae, inhabits Zrmanja River, and this species together with Squalius illyricus and Squalius sp. occurs in the Krka River drainage.

The data presented above on the distribution of Squalius chubs in the northern Adriatic Basin support the assumption by Bogutskaya and Zupančič (2010) that the range of Squalius janae encompassing most of Istra Peninsula, except for its north-western section, is determined by the geology of two flysch basins forming the base of the Istra Peninsula. These basins are the Trieste Flysch Basin and the Pazin Flysch Basin bordered by limestone areas of the Buje and West Istria anticlines in the west and the Čičarja and Učka mountain ranges, which belong to the Dinaric Alps, in the east (Fišer et al. 2006; Babić et al. 2007). It is well known and discussed in the literature (e.g. Lindberg 1972; Bianco and Miller 1990; Holčík and Mrakovčić 1997) that during several marine regressions including the most recent ones, for example during the last Würm glacial maximum, rivers of both slopes of the Adriatic basin used to form an extensive drainage of the Palaeo-Po-Isonzo (e.g. CLIMAP 1976, Rodić 1981: fig. 21). However, the distribution pattern of different fish species, the occurrence of a certain number of endemic species in particular, does not support a simple model of fish dispersal within the Palaeo-Po’s tributaries. Indeed, the distributional pattern of most fish taxa stands against a hypothesis that assumes a complete Palaeo-Po habitat connectivity and indicates a disrupted surface palaeohydrography that was heavily fragmented by karstification in the whole Dinaric area. A similar assumption has been made for a number of taxa other than fish inhabiting the Dinaric Karst (Sket 2002, Trontelj et al. 2007).

### Key to Squalius species occurring in Slovenia and north-western Croatia including Zrmanja

**Table:** 

1.	Lower jaw length less than operculum depth; 5th infraorbital narrow (deeper than wide) or absent	Squalius zrmanjae
–	Lower jaw length exceeding operculum depth; 5th infraorbital extensive (considerably wider than deep), covering together with large 4th infraorbital most part of outer surface of the musculus dilatatoris operculi	2
2.	Commonly 8½ branched anal-fin rays; pelvic and anal fins red or orange with no black pigment	Squalius cephalus
–	Commonly 9½ branched anal-fin rays; pelvic and anal fins slightly red, orange or yellow with black pigment of varying intensity	3
3.	Conspicuous silvery tint in life colouration; scales easily lost; iris, pectoral, pelvic and anal fin pigmentation with yellow shades; head length 27–32% SL, lower jaw length exceeding caudal peduncle depth; mouth cleft straight, oblique	Squalius janae
–	No silvery tint in life colouration, overall colouration with brownish or bronze tones; scales not easily lost; iris, pectoral, pelvic and anal fin pigmentation with reddish or orange shades; head length 26–29% SL, lower jaw length equal to or shorter than caudal peduncle depth; slightly curved mouth cleft	Squalius squalus
